# Renin Angiotensin System as a Regulator of Cell Volume. Implications to Myocardial Ischemia

**DOI:** 10.2174/157340309787048149

**Published:** 2009-01

**Authors:** Walmor C De Mello

**Affiliations:** Medical Sciences Campus, School of Medicine, UPR, San Juan, PR 00936-5067, USA

**Keywords:** Cell volume, myocardial ischemia, renin, angiotensin II, angiotensin (1-7) intracrine, stretch.

## Abstract

It is known that long lasting changes in cell volume are incompatible with cellular functions. In the present review, I discussed the role of cell volume on gene expression and protein synthesis as well as the importance of the renin angiotensin system on the regulation of cell volume in the failing heart. Moreover, the relationship between mechanical stretch, cell volume and the renin angiotensin system as well some translational studies are also described and their relevance to the prevention or reduction of cardiac damage during myocardial ischemia is emphasized.

## INTRODUCTION

Animal cells are surrounded by a membrane highly permeable to water [1] and osmosis is an important cause of water transport across the surface cell membrane generating changes in cell volume. It is well known that long lasting variations in cell volume are incompatible with cell functions and consequently, the preservation of normal cell volume is of fundamental importance to cell biology. This difficult task is accomplished through complex mechanisms including two major ionic transport systems: a) the Na-K—2Cl cotransport [2] and b) the Na/H exchanger [3] which alkalizes the cell with consequent activation of Cl/HCO_3_ exchanger. Variations in cell volume cause important changes in cellular functions like the activation stretch-sensitive ion channels, change in metabolism, gene expression and protein synthesis [4, 5]. Hypotonic stress induced by ischemia, for instance, leads to accumulation of metabolites intracellularly with consequent cell swelling due to water entering the cell. Moreover, long-lasting myocardial ischemia causes profound changes in cellular physiology including alteration in genetic expression as evidenced by the finding that hypertonic solution or high ionic strength stimulates the expression of aldose reductase and the Na^+^-coupled transport systems for several amino acids which increase intracellular osmolarity counteracting the enhanced extracellular osmotic pressure [6]. Cell shrinkage increases the expression of heat shock proteins and of other proteins such as P-glycoprotein, ClC-K1, and Na^+^-K^+^-ATPase α_ 1_-subunit, cyclooxygenase-2, the GTPase-activating protein for Rac α_1_-chimerin, the immediate early gene transcription factors Egr1-1 and c-Fos, vasopressin, phospho*enol*pyruvate carboxykinase, tyrosine aminotransferase, tyrosine hydroxylase, dopamine β-hydroxylase, matrix metalloproteinase 9, and several matrix proteins [7]. Cell swelling, on the other hand, increases the expression of proteins like β-actin, tubulin, cyclooxygenase-2, extracellular signal-regulated kinases ERK-1 and ERK-2, JNK, the transcription factors c-Jun and c-Fos, ornithine decarboxylase, and tissue plasminogen activator [7]. However, our knowledge of the mechanisms involved in the changes in gene expression is meager [8].

## RENIN ANGIOTENSIN SYSTEM REGULATES THE HEART VOLUME. INTRACRINE *VS*. EXTRACELLULAR RENIN AND ANGIOTENSIN II

Evidence is available that the plasma renin angiotensin system is involved in the regulation of blood volume and arterial blood pressure and that there is a local renin angiotensin system in the heart which promotes cardiac remodeling, changes in cell communication and inward calcium current in the normal and in the failing heart [9 -11]. In addition, angiotensin II and renin dialyzed into the cell, causes cell uncoupling - an effect suppressed by ACE inhibitors and AT1 blockers [9,10] supporting the view that there is an intracellular renin angiotensin system [9-12]. Recent observations [13] indicated that the renin angiotensin aldosterone system is involved in the regulation of cell volume in the normal and particularly in the failing heart. Indeed, extracellular renin or Ang II increases the cell volume in normal and failing heart through the inhibition of the sodium pump and the activation of the Na-K-2Cl cotransporter [13] while intracellular renin and Ang II reduces the cell volume by enhancing the electrogenic sodium- potassium pump [13]. This is a finding of seminal importance to heart cell biology because alterations of cell volume induce the release of ATP, hormones like insulin and renin, neurotransmitters [7] and activates plasma membrane receptors and integrins which also participate in the regulation of cell volume [1]. On the other hand, cell volume regulation following cell swelling involves the efflux of ions through activation of K+ channels and or anion channels and parallel activation of K+/H+ exchange and Cl/HCO_3_ exchange while cell shrinkage causes accumulation of ions through different mechanisms including activation of the Na-K-2Cl cotransporter and Na+/H+ exchanger [7]. It is then conceivable that intracellular renin and Ang II play an important role re-establishing the cell volume increased during normal or pathological conditions. Indeed, it is recognized that alteration of cell volume regulation contributes to several diseases such as diabetic ketoacidosis, liver insufficiency, sickle cell anemia and infection [7] and that cell swelling-activated Cl current (_ICl swell_), which is broadly distributed throughout the heart, shortens the action potential, depolarizes the cell membrane, and is a potential cause of cardiac arrhythmias [14]. The presence of a renin transcript that does not encode a secretory signal [15] and is over-expressed during myocardial infarction, raises the possibility that intracellular renin has an important role in the regulation of heart cell volume which is relevant during myocardial ischemia [27].

## MECHANICAL STRETCH, CELL VOLUME AND RENIN ANGIOTENSIN SYSTEM

During heart failure, ventricular hypertrophy or dilatation as that seen in dilated cardiomyopathy, elicits mechanical stress with concurrent changes of extracellular matrix and cytoskeleton [16,17]. On the other hand, I_Cl,swell _which is implicated in the regulation of cell volume in all types of cells [18], is activated by cell swelling during ischemia/reperfusion [14,19-21]. Myocardial ischemia which generates mechanical forces and deformation of cell membrane [14,] with consequent increase of tension of surface cell membrane, activates several ionic channels enhancing their open probability without altering single channel conductance [18]. Mechanical stress or cell swelling also stimulates protein kinase C [22, 23] and increases tyrosine phosphorylation of several proteins [7], a finding particularly relevant because it is known that Ang II changes the inward calcium current in the heart through the activation of PKC and tyrosine kinases [24]. Membrane stretch and ionic channel activation might involve: a) the release of fatty acids from the membrane and activation of stretch-sensitive channels; b) stretch activation of some component of the cytoskeleton such as spectrin [see 7]. It is known that stretching depolarizes the heart cell membrane during diastole, changes the action potential and produces arrhythmias [25] and that part of these effects are mediated by stretch-activated ion channels [25]. Interestingly, mechanical stress activates angiotensin II AT1 receptors independently of angiotensin II [26] suggesting that the AT1 receptor is a mechanical sensor by itself or it is associated with stretch sensors such as integrins [26]. Because chronic deformation of surface cell membrane like that seen in heart failure, essential hypertension and myocardial ischemia causes mechanical stress, alteration of genetic expression and changes in heart cell excitability are likely events which might be in part associated with AT1 receptor activation [28].

## INTRACELLULAR *VS*. EXTRACELLULAR RENIN, ANGIOTENSIN II AND CELL VOLUME

**Fig. (1):**
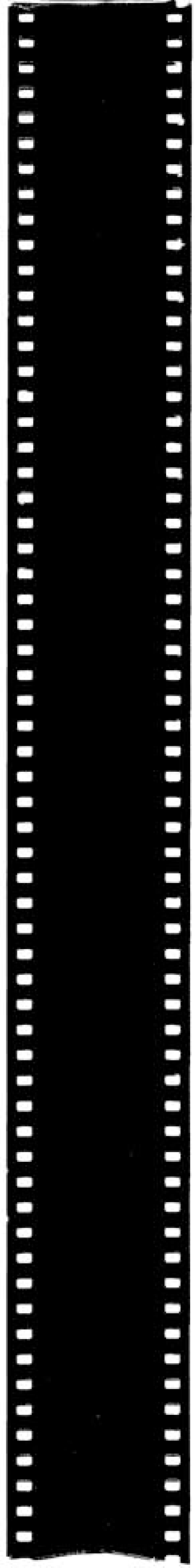
Diagram illustrating the influence of myocardial ischemia on heart cell volume, the potentiating effect of plasma RAS activation and the influence of the intracellular renin and angiotensin II as well as extracellular angiotensin (1-7) on cell volume.

Of particular interest was the recent finding that intracellular dialysis of Ang II in myocytes isolated from the failing heart, reverses the increase of cell volume elicited by hypotonic solution [13] raising the possibility that the activation of the intracellular renin angiotensin system [11] be beneficial during myocardial ischemia by reducing cell volume. Consequently, there is a decrease of the activation of I_Cl,swell _and prevention of action potential shortening, an important cause of cardiac arrhythmias. Interestingly, extracellular renin and Ang II have opposite effects on cell volume inhibiting the sodium pump and activating the Na-K-2Cl cotransporter with consequent increase of cell volume [13]. These findings support the view that the renin angiotensin system is involved in the regulation of cell volume and that the activation of plasma renin angiotensin system is harmful during ischemia/reperfusion when the cell volume is already increased (Fig. **1**). Indeed, it is known that angiotensin II contributes to heart damage during ischemia/ reperfusion and that Ang II AT1 receptor blockers as well as ACE inhibitors have protective effects in experimental animal models of myocardial ischemia as well as in humans [28]. Since evidence is available that the Na-K-2 Cl cotrasporter is constantly activated during heart failure [14], the question remains if there is a relation between the activation of the renin angiotensin system and the activation of the cotransporter. If this is the case, the beneficial effect of ACE inhibitors and angiotensin II AT1 receptor blockers during myocardial ischemia is related, at least in part, to the suppression of the effect of angiotensin II on cell volume.

## ACE2, ANGIOTENSIN (1-7) AND CELL VOLUME ?

Angiotensin II, however, is hydrolyzed to angiotensin (1-7) by ACE2 [29] with consequent the generation of angiotensin (1-7), which counteracts some harmful effects of Ang II [30] including the block of impulse propagation seen during ischemia/reperfusion. The beneficial effect of angiotensin (1-7) on impulse propagation is related to the activation of the sodium pump and hyperpolarization of cell membrane [31]. Since ACE2 is over-expressed during heart failure [32, 33], one wonders if the enhanced expression of ACE2 is involved on the regulation of heart cell volume. This hypothesis seems to be supported by recent findings that angiotensin (1-7) increments the sodium pump and reduces the cell volume by 15% within 25 minutes in the failing heart [De Mello, unpublished]. It is then conceivable that the improvement of cardiac function and decreased incidence of cardiac arrhythmias during ischemia/reperfusion elicited by Ang (1-7), be related, at least in part, to the decrease of heart cell volume (Fig. **1**). According to these findings, the balance between ACE and ACE2 expression seems to be a determinant factor on the regulation of cell function and cell volume and that the beneficial effect of ACE inhibitors or AT1 receptor blockers during myocardial ischemia and heart failure be related to the prevention of cell swelling induced by extracellular renin and angiotensin II. On the other hand, intracellular renin and angiotensin II might be beneficial by reducing the cell swelling.

## CARDIOPROTECTION AGAINST ISCHEMIA/REPERFUSION INJURY. TRANSLATIONAL STUDIES

Translational studies are of fundamental importance for the prevention or treatment of the harmful effects of myocardial ischemia including the changes in cellular volume. Currently, two major strategies have being tried: a) continuous promotion of cardioprotective factors; b) treatment of cardiac damage. Adenosine and KATP channel opener nicorandil ameliorate reperfusion injury in the clinical setting [34]. Concerning gene therapy, a cardioprotective effect has been successfully obtained through genetic modulation of PKC-epsilon [35] and of NO synthase [36].As emphasized by different authors, the major problem is to identify the target clinical population for these therapies before symptoms and signs are develop in order to prevent heart damage. Tumor necrosis factor (TNF), for instance, is enhanced in myocardial tissue after ischemia/reperfusion and it is known to induce preinflammatory signaling. Indeed, in humans, there is a direct correlation between circulating levels of TNF and functional capacity and survival [37] what suggests that, at least in male, blockade of 55-kDA TNF receptor be beneficial during ischemia/reperfusion. Future translational studies will certainly provide the appropriate target clinical population for each of these alternatives and will offer new avenues for the prevention of cardiac damage during ischemia reperfusion and the consequent change in heart cell volume.
